# Structural enzymology using X-ray free electron lasers

**DOI:** 10.1063/1.4972069

**Published:** 2016-12-15

**Authors:** Christopher Kupitz, Jose L. Olmos, Mark Holl, Lee Tremblay, Kanupriya Pande, Suraj Pandey, Dominik Oberthür, Mark Hunter, Mengning Liang, Andrew Aquila, Jason Tenboer, George Calvey, Andrea Katz, Yujie Chen, Max O. Wiedorn, Juraj Knoska, Alke Meents, Valerio Majriani, Tyler Norwood, Ishwor Poudyal, Thomas Grant, Mitchell D. Miller, Weijun Xu, Aleksandra Tolstikova, Andrew Morgan, Markus Metz, Jose M. Martin-Garcia, James D. Zook, Shatabdi Roy-Chowdhury, Jesse Coe, Nirupa Nagaratnam, Domingo Meza, Raimund Fromme, Shibom Basu, Matthias Frank, Thomas White, Anton Barty, Sasa Bajt, Oleksandr Yefanov, Henry N. Chapman, Nadia Zatsepin, Garrett Nelson, Uwe Weierstall, John Spence, Peter Schwander, Lois Pollack, Petra Fromme, Abbas Ourmazd, George N. Phillips, Marius Schmidt

**Affiliations:** 1 Physics Department, University of Wisconsin-Milwaukee, 3135 N. Maryland Ave, Milwaukee, Wisconsin 53211, USA; 2 Department of BioSciences, Rice University, 6100 Main Street, Houston, Texas 77005, USA; 3 Department of Physics, Arizona State University, Tempe, Arizona 85287, USA; 4 Marbles Inc., 1900 Belvedere Pl, Westfield, Indiana 46074, USA; 5 Center for Free-Electron Laser Science, DESY, Notkestrasse 85, 22607 Hamburg, Germany; 6 University of Hamburg, Luruper Chaussee 149, 22761 Hamburg, Germany; 7 Linac Coherent Light Source, Stanford Linear Accelerator Center (SLAC) National Accelerator Laboratory, 2575 Sand Hill Road, Menlo Park, California 94025, USA; 8 Department of Applied and Engineering Physics, Cornell University, 254 Clark Hall, Ithaca, New York 14853, USA; 9 Hauptman-Woodward Institute, State University of New York at Buffalo, 700 Ellicott Street, Buffalo, New York 14203, USA; 10 School of Molecular Sciences and Biodesign Center for Applied Structural Discovery, Arizona State University, Tempe, Arizona 85287-1604, USA; 11 Lawrence Livermore National Laboratory, Livermore, California 94550, USA

## Abstract

Mix-and-inject serial crystallography (MISC) is a technique designed to image enzyme catalyzed reactions in which small protein crystals are mixed with a substrate just prior to being probed by an X-ray pulse. This approach offers several advantages over flow cell studies. It provides (i) room temperature structures at near atomic resolution, (ii) time resolution ranging from microseconds to seconds, and (iii) convenient reaction initiation. It outruns radiation damage by using femtosecond X-ray pulses allowing damage and chemistry to be separated. Here, we demonstrate that MISC is feasible at an X-ray free electron laser by studying the reaction of *M. tuberculosis* ß-lactamase microcrystals with ceftriaxone antibiotic solution. Electron density maps of the *apo*-ß-lactamase and of the ceftriaxone bound form were obtained at 2.8 Å and 2.4 Å resolution, respectively. These results pave the way to study cyclic and non-cyclic reactions and represent a new field of time-resolved structural dynamics for numerous substrate-triggered biological reactions.

## INTRODUCTION

ß-lactams are a class of antibiotics used frequently in the treatment of bacterial infection.^1^ They form one of the most important antibiotic families, including a broad range of molecules such as penicillin derivatives, cephalosporins, and ß-lactamase inhibitors, as well as penems and carbapenems.^2–4^ The broad range of activity of these antibiotics against both Gram-negative and Gram-positive pathogens renders ß-lactams some of the most widely used antibiotics worldwide.^5^ Evolutionary bacterial response has given rise to ß-lactamase enzymes able to hydrolyze the ß-lactam ring, thereby conferring antibiotic resistance to bacteria.^4,6^ This increasing resistance now represents a serious challenge for the efficacy of this class of antibiotics.^7^

Tuberculosis (TB), a disease caused by the bacteria *Mycobacterium tuberculosis* (MTb), is a major health concern worldwide with approximately 9.6 × 10^6^ new cases in 2014, 1.5 × 10^6^ deaths in the same year. TB remains a leading cause of death among AIDS patients.^8^ One reason tuberculosis has evaded treatment with β-lactams is the evolution of a singular ß-lactamase (BlaC) found in MTb.^9,10^ Understanding the structural origins of binding modes of BlaC has resulted in improved compounds for the treatment of not only TB but also Extensively Drug Resistant (XDR-TB) strains of the disease.^11^

BlaC catalyzes the opening of the ß-lactam ring by nucleophilic attack via an active site serine residue that generates an acyl-enzyme intermediate state. The intermediate formation is followed by the generation of the ring-opened, inactive product through hydrolysis of the ester bond.^12^ In addition, in cephalosporin substrates, the thiodioxotriazine moiety is a good leaving group and likely dissociates in a concerted manner with the ring opening and formation of the acyl intermediate.^13–15^ Previous studies have further shown that BlaC is a broad-spectrum ß-lactamase, hydrolyzing virtually all ß-lactam classes.^16^ Direct observations of this process would lead to a better understanding of enzyme-drug interaction and could allow for novel ways to bypass or inhibit the BlaC catalyzed ring hydrolysis, such as drug targeting of stable intermediate states. Many structures of *M. tuberculosis* BlaC have been solved with various substrates via conventional X-ray crystallography. However, these are static structures showing only the binding interactions and not the coordinated actions of the enzyme that result in binding and catalysis.^17–20^

Time-resolved crystallography^21^ enables time-dependent structure determination to reveal protein kinetics.^22^ Light-triggered studies at synchrotrons can provide up to 100 ps time resolution.^23–25^ However, one of the major challenges with time-resolved macromolecular crystallography is the study of reactions that do not “reset” for the next X-ray pulse. Reactions that are not light-driven, like most enzymatic reactions, can be difficult or impossible to capture in crystals because of the difficulty in initiating reactions in a concerted way in the molecules throughout the volume of a large crystal needed for conventional crystallographic measurements. Calculations show that this issue can be resolved using micron-sized crystals because the reduced size drastically decreases the diffusion time of the substrate into the crystal.^26–28^ If diffusion times are much faster than the enzymatic turnover times, this provides an elegant and straightforward way to initiate reactions in enzyme crystals.

Crystals of this small size (<10 *μ*m) are difficult to examine at a synchrotron, due to radiation damage and the lack of well-established sample delivery methods. The introduction of X-ray free electron lasers (XFELs) therefore presents new opportunities in several regards. XFELs have enabled time-resolved structure determination of fast light-activated proteins after optical illumination, allowing structures of protein conformational changes to be imaged on time scales of hundreds of femtoseconds that have been previously impossible to reach.^29–33^ However, light sensitive proteins are only one type of proteins whose dynamics are of interest. Many, if not most, physiologically relevant reactions are initiated chemically rather than by light. The ability to mix a protein with a substrate and directly observe the various structural intermediates associated with a chemical reaction would open an entirely new field in structural enzymology, incorporating a new, more holistic perspective on substrate binding, significant intermediates, bound products, and the kinetics of their appearance and disappearance. Such reactions need to be studied at room temperature in order to explore relevant kinetics and discover the structure of the transitional states. In this paper, we present the evidence that such experiments are possible, using the mix-and-inject serial crystallography (MISC) approach at an XFEL. During the review of this paper, Stagno *et al.* also reported successful diffusion of an amino acid (adenine) into RNA with a 10 s mixing time using a similar setup, providing further credence that the technique is a viable method for future enzymological studies.^34^

## RESULTS AND DISCUSSION

**FIG. 1.**
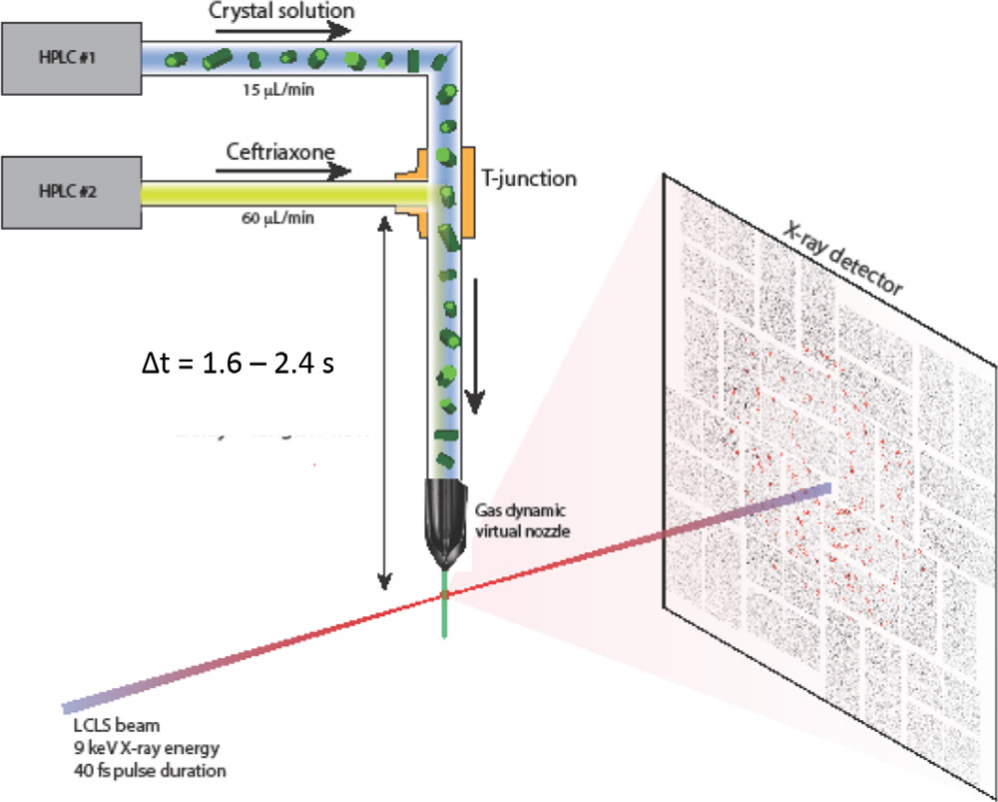
Data collection schematic showing the T-junction set-up used for a mixing time of about 2 s. The T-junction was placed outside of the nozzle rod in our experiment but could also be engineered to fit inside closer to the interaction region for shorter mixing times.

Structures of BlaC with and without ceftriaxone (CEF), a cephalosporin antibiotic substrate, were measured at room temperature using the Coherent X-ray Imaging (CXI) instrument at the Linac Coherent Light Source (LCLS)^35^ at SLAC. The antibiotic was mixed with microcrystals of BlaC using a T-junction (Fig. 1), which allowed for a diffusion/reaction time of about 2 s (see supplementary material). The time-resolution of a mix-and-inject experiment also depends on the rate of diffusion of the substrate into the crystals, limiting the time resolution that can be achieved at synchrotron sources.^26^ Our crystals have average widths on the order of 3–10 *μ*m in two of the dimensions and thickness of 2–3 *μ*m in the third. Diffusion calculations indicate that the diffusion of the substrate into these microcrystals takes only about 1–15 ms.^26^ This is about one hundred times smaller than the time required for the crystal substrate mixture to travel to the injector and can be neglected in our experiment. Therefore, with improved mixing-injectors,^36,37^ this time scale should allow for a variety of enzyme reactions to be studied, from the long time delays (seconds) to short time delays (500 *μ*s).^37^ To demonstrate substrate binding with MISC as a proof-of-principle, two separate room temperature data sets were collected using the serial femtosecond crystallographic (SFX) approach: an *apo* structure from 12 853 indexed diffraction patterns and a “mixed” structure from 22 646 indexed patterns with CEF added.

**FIG. 2.**
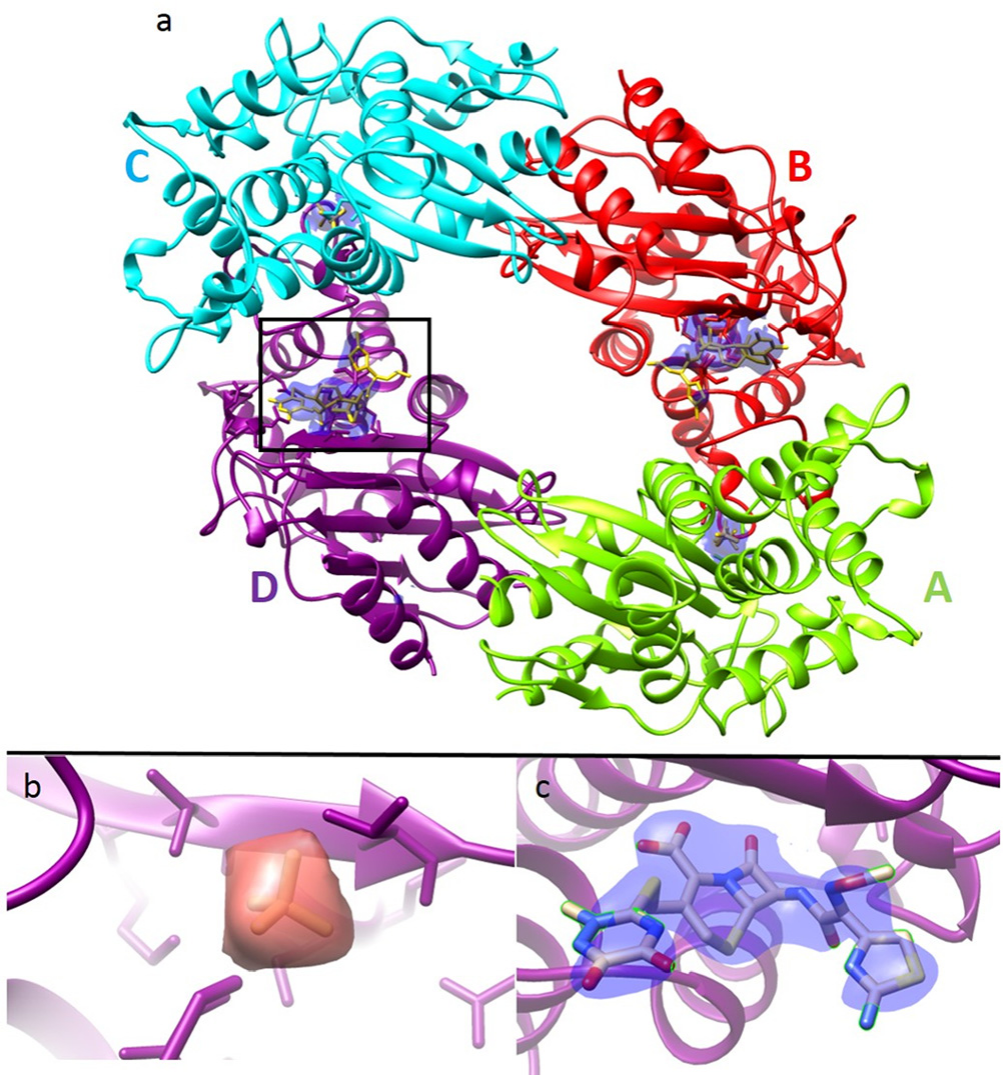
Electron density in the catalytic cleft of BlaC. (a) Refined model of the entire tetramer (σ = 1.1) in the asymmetric unit after mixing. The mixed electron density (2F_o_-F_c_) is shown in blue in the binding pockets. Subunits A and C contain phosphate while subunits B and D have a bound ceftriaxone, with the electron density of D being slightly stronger. (b) Enlarged section of subunit D showing the unmixed ED, which corresponds to a bound phosphate. (c) Enlarged section of subunit D showing the mixed ED (blue electron density) with ceftriaxone modelled in. Slightly different views of the same subunit binding pocket are shown in (b) and (c); however, there are minimal changes to the ligand binding sphere (see supplementary material, Table S2).

The data collected were used to determine the structures to a resolution of 2.8 Å for the *apo* and 2.4 Å for the mixed form. Fig. 2 shows an overview of the BlaC asymmetric unit, as well as the ligand binding pocket from both the *apo* and the BlaC mixed with CEF. The subunits labeled A and C show no binding to ceftriaxone, highlighting the point that the kinetics of a catalytic cycle in the crystal are not necessarily the same as in solution. The crystal packing of these subunits and their crystal contacts appear to prevent facile access to the binding pocket, showing only the presence of phosphate. The density in the binding pocket of subunits D shows strong evidence of substrate accessibility with the active site electron density (ED) from subunit B being less prominent, but still present. Given the long mixing times of this experiment along with the measured K(cat) for ceftriaxone of 49 ± 17/min (turn over time of 1.22 s),^16^ it is likely that on average, at least one CEF molecule, perhaps two, was cleaved during the time between mixing and actually interacting with the X-rays. Models of the uncleaved, acyl intermediate, and product complex of the ceftriaxone molecule were modelled to attempt to fit the electron density (ED) found in the BlaC active site. For the current time point of 1.4–2.6 s, we expect to observe the superposition of several chemical intermediates in the ED. Since our time delay is more than 2 s and the turnover time is on the order of seconds, a steady state has most likely formed. The steady state consists of all states along the enzymatic pathway that are occupied according to their free energies. The enzyme substrate complex, any putative intermediate, and the product state may all be occupied and represented by the average electron density. Therefore, we attempted to refine three different structures into the electron density (Figs. S3–S5, supplementary material). Accordingly, the chemical form with the longest presence in the active site, i.e., the rate-limiting step, will be most represented in the ED. From the modelling of our data, it appears that we mainly observe a steady state. The K_m_ value was previously measured at 520 *μ*M.^16^ When considered in conjunction with the moderately slow turnover rate of approximately 1.22 s, the data support that the rate-limiting step for this reaction has not been measured but is likely the formation of the acyl intermediate (for a further discussion of the chemical kinetics see the supplementary material). In this state, the enzyme requires time to achieve coordinated alignment for the nucleophilic attack of the Ser70 residue on the β-lactam ring before the reaction can proceed to the acyl intermediate. As seen in other inhibitor complexes, other protein interactions we observe with ceftriaxone (and the phosphate) include threonine (Thr237), glutamic acid (Glu166), lysine (Lys73), and an additional serine residue (Ser128).

We next compare the active site structure presented here with the structure when the antibiotic cefamandole is soaked into large BlaC crystals (PDB code 3N8S^38^). The structure of the Michaelis complex as well as the covalent acyl intermediate for mutants of BlaC was solved at a synchrotron at cryogenic temperatures.^38^ In the cefamandole structure, the Ser70 nucleophilic oxygen is 2.8 Å from the β-lactam ring carbonyl carbon compared to 3.2 Å for the ceftriaxone Ser70 oxygen. Both the complexes are positioned almost identically in the active site. Interestingly, the Ser142, in our structure, which binds the carboxylate group, is turned and the carboxylate is positioned over 3 Å away. This suggests that the ceftriaxone molecule is bound more loosely and has a lower affinity in the active site than cefamandole; however, it is impossible to fully separate the differences between the two due to the differences in techniques used to solve the structure, especially the difference between room-temperature and cryo-cooled data collection. For Thr253 and K250, this results in the ceftriaxone carboxylate group being rotated by nearly 90°. These observations explain the difference between the observed kinetic parameters of cefamandole (K_m_ = 184 *μ*M with kcat = 3500 min^−1^) and ceftriaxone (K_m_ = 520 *μ*M and k_cat_ = 49 min^−1^).^16^ The high turnover rate of cefamandole is why it was deemed unsuitable for this initial proof-of-concept work. Since we see no clear density connecting the Ser70 to the lactam ring, we have modeled the structure as the enzyme-substrate complex in this study (see Fig. 2); however, it is most likely a mixture of free enzyme, enzyme substrate complex, and product bound to the enzyme (see supplementary material for further discussion).

In conclusion, our data show clear electron density in the binding pockets, which changes between the *apo* and mixed states. The electron density of the mixed sample matches ceftriaxone and its products fairly well, which shows that binding of the substrate to BlaC in the crystal took place, and occurred extensively across the crystals so that electron density is clearly visible in the active site. The lack of strong density for the thiodioxotriazine ring also suggests that the enzyme is turning over, although we cannot rule out the possibility that it is just disordered in this context. This constitutes a proof-of-principle that room temperature MISC on enzymes is possible. Future experiments with further increase in time resolution of the mixing process should allow us to separate the events in time throughout the course of the catalytic cycle, free of radiation-damage and photoelectron reduction effects. The potential impact of this method on the field of enzymology and related disciplines is unquestionably exciting, since it allows both the atomic structures of stable intermediates and the time scales of their inter-conversions to be observed directly at atomic resolution.

## SUPPLEMENTARY MATERIAL

See supplementary material for the further detailed discussion and analysis.
